# Left ventricular long axis strain: a new prognosticator in non-ischemic dilated cardiomyopathy?

**DOI:** 10.1186/s12968-016-0255-0

**Published:** 2016-06-07

**Authors:** Johannes H. Riffel, Marius G. P. Keller, Franziska Rost, Nisha Arenja, Florian Andre, Fabian aus dem Siepen, Thomas Fritz, Philipp Ehlermann, Tobias Taeger, Lutz Frankenstein, Benjamin Meder, Hugo A. Katus, Sebastian J. Buss

**Affiliations:** Department of Cardiology, University of Heidelberg, INF 410, 69120 Heidelberg, Germany; DZHK (German Centre for Cardiovascular Research), partner site, Heidelberg, Germany; Department of Cardiology, Angiology and Pneumology, University of Heidelberg, Im Neuenheimer Feld 410, 69120 Heidelberg, Germany

**Keywords:** Dilated cardiomyopathy, Cardiovascular magnetic resonance, Prognosis, Left ventricular function, Long axis strain

## Abstract

**Background:**

Long axis strain (LAS) has been shown to be a fast assessable parameter representing global left ventricular (LV) longitudinal function in cardiovascular magnetic resonance (CMR). However, the prognostic value of LAS in cardiomyopathies with reduced left ventricular ejection fraction (LVEF) has not been evaluated yet.

**Methods and results:**

In 146 subjects with non-ischemic dilated cardiomyopathy (NIDCM, LVEF ≤45 %) LAS was assessed retrospectively from standard non-contrast SSFP cine sequences by measuring the distance between the epicardial border of the left ventricular apex and the midpoint of a line connecting the origins of the mitral valve leaflets in end-systole and end-diastole. The final values were calculated according to the strain formula.

The primary endpoint of the study was defined as a combination of cardiac death, heart transplantation or aborted sudden cardiac death and occurred in 24 subjects during follow-up. Patients with LAS values > −5 % showed a significant higher rate of cardiac events independent of the presence of late gadolinium enhancement (LGE). The multivariate Cox regression analysis revealed that LVEDV/BSA (HR: 1.01, *p* < 0.05), presence of LGE (HR: 2.51, *p* < 0.05) and LAS (HR: 1.28, *p* < 0.05) were independent predictors for cardiac events. In a sequential cox regression analysis LAS offered significant incremental information (*p* < 0.05) for the prediction of outcome in addition to LGE and LVEDV/BSA. Using a dichotomous three point scoring model for risk stratification, including LVEF <35 %, LAS > −10 % and the presence of LGE, patients with 3 points had a significantly higher risk for cardiac events than those with 2 or less points.

**Conclusion:**

Assessment of long axis function with LAS offers significant incremental information for the prediction of cardiac events in NIDCM and improves risk stratification beyond established CMR parameters.

## Background

Non-ischemic dilated cardiomyopathy (NIDCM) is the second most common aetiology of heart failure and a leading indication for heart transplantation [1, 2]. Although there has been a significant improvement in prognosis in NIDCM over the last decades, mortality is still high and an early diagnosis and risk stratification is crucial [3]. The main features of NIDCM are left ventricular dilatation and systolic dysfunction, while patients present with heterogeneous symptoms, ranging from asymptomatic patients to terminal heart failure [4]. Classical risk factors, which are associated with a poor prognosis, include age and male gender, NYHA class, impaired left ventricular ejection fraction (LVEF) and specific cardiac biomarkers [5–7]. Moreover, presence of fibroses, which can be seen in about 30 % of the patients with NIDCM, is associated with poor prognosis [8–10].

Due to its excellent intrinsic blood-to-tissue contrast and high reproducibility, cardiovascular magnetic resonance (CMR) is nowadays considered as the non-invasive gold standard for the evaluation of left ventricular (LV) function [11]. Additionally, assessment of late gadolinium enhancement (LGE) permits diagnostic classification and risk stratification in patients with NIDCM [8, 12–15].

The assessment of myocardial strain provides additional prognostic value in heart failure patients [16–18]. Lately, it has been shown that measurement of cardiac strain with feature tracking software (FTI) provides incremental prognostic information in NIDCM patients [19].

Lately, we established long axis strain (LAS) in CMR as a reliable and fast assessable parameter for LV global longitudinal function which provides high sensitivity and specificity in discriminating patients with cardiomyopathies from healthy subjects without the necessity of any additional offline deformation analysis software tools and without application of gadolinium contrast agents. Moreover, LAS analysis shows good inter- and intraobserver agreement and can be performed much faster than FTI derived strain analysis [20]. However, until now there exist no data about the prognostic value of LAS analysis in cardiomyopathies with reduced LVEF.

The aim of the present study was to evaluate whether LAS assessed with CMR offers prognostic information besides standard CMR parameters in patients with NIDCM.

## Methods

### Study population

Our study population consisted initially of 210 consecutive patients with NIDCM who were prospectively included in our study after referral to the ‘Cardiomyopathy Center’ at the University Hospital Heidelberg between May 2005 and November 2009. 64 patients had an LVEF over 45 % and were therefore excluded. LAS was analyzed retrospectively in 146 patients. A part of the population was previously reported [19]. CMR was performed as part of our standard institutional protocol for the evaluation of cardiomyopathies, unless one of the following contraindications for CMR was present: cardiac pacemaker or implantable cardioverter defibrillator (ICD), other CMR incompatible metallic implants, severe claustrophobia, obesity preventing patient to enter the scanner bore, pregnancy and lactation. Chronic renal failure with an estimated GFR <30 ml/min/1.73 m^2^ was added as an exclusion criterion for administration of intravenous CMR contrast agents after July 2007.

The diagnosis of NIDCM was based on the 1995 World Health Organization/International Society and Federation of Cardiology criteria [21]. Inclusion criteria for the current retrospective analysis were: impaired systolic function (LVEF ≤45 %) assessed with CMR and absence of (i) significant coronary artery disease (defined as ≥50 % luminal stenosis in one coronary artery) by coronary angiography, previous coronary revascularization or myocardial infarction, (ii) valvular disease, (iii) hypertensive heart disease and (iv) congenital abnormalities. All patients had congestive heart failure and were examined in a clinically stable condition (NYHA functional class <III). All patients gave their informed consent and our investigation was carried out after approval by the local Ethics Committee of the University of Heidelberg and in accordance with the Declaration of Helsinki.

### Follow-up data and definition of study endpoints

Cardiac death, heart transplantation and sudden cardiac death aborted by appropriate ICD discharge due to ventricular tachycardia or fibrillation were defined as hard cardiac events building the *primary endpoint* of our study. Cardiac events together with the occurrence of hospitalization due to congestive heart failure were used as a *secondary endpoint*. In case of patients undergoing heart transplantation, the follow-up data was censored at the time of transplantation. In case of several simultaneous cardiac events per patient, the worst event was selected (cardiac death > transplantation > aborted SCD due to appropriate ICD shock > hospitalization due to heart failure). Otherwise, only the first event for each patient was included in the analysis for composite end-points. Personnel unaware of the CMR results contacted each subject or an immediate family member for acquisition of the follow-up data.

### CMR acquisition and analysis

Standard CMR was performed on a 1.5T clinical scanner (Achieva®, Philips Healthcare, Best, The Netherlands) equipped with a cardiac phased array receiver coil. Cine images were obtained using a breath-hold segmented-k-space balanced fast-field echo sequence (SSFP) employing retrospective ECG gating in long axis planes (2, 4 and 3 chamber views) as well as in contiguous short axis slices covering the whole ventricles from the annulus of the atrioventricular valves to the apex, with 35 phases per cardiac cycle. All analyses were performed on a commercially available workstation (Viewforum®, Philips Healthcare). Results for ventricular volumes, ejection fraction and LV myocardial mass were derived from short axis slices. The presence of late gadolinium enhancement was evaluated by two independent observers experienced in CMR, who were blinded to clinical data and outcome. To exclude artefact, LGE was deemed present only if visible in two orthogonal views. Left ventricular global function index (LVGFI) was assessed as described previously [22].

### Assessment of LAS

Values for LAS were assessed in 2 and 4 chamber views by measuring displacement of the mitral annulus. The distance between the epicardial border of the LV apex and the middle of a line connecting the origins of the mitral valve leaflets was measured in both end-systole and end-diastole. The value in percentage for LAS was finally determined according to the strain formula:

$$ LAS=\frac{lengt{h}_{end- systole}- lengt{h}_{end- diastole}}{lengt{h}_{end- diastole}}\ast 100 $$

**Fig. 1:**
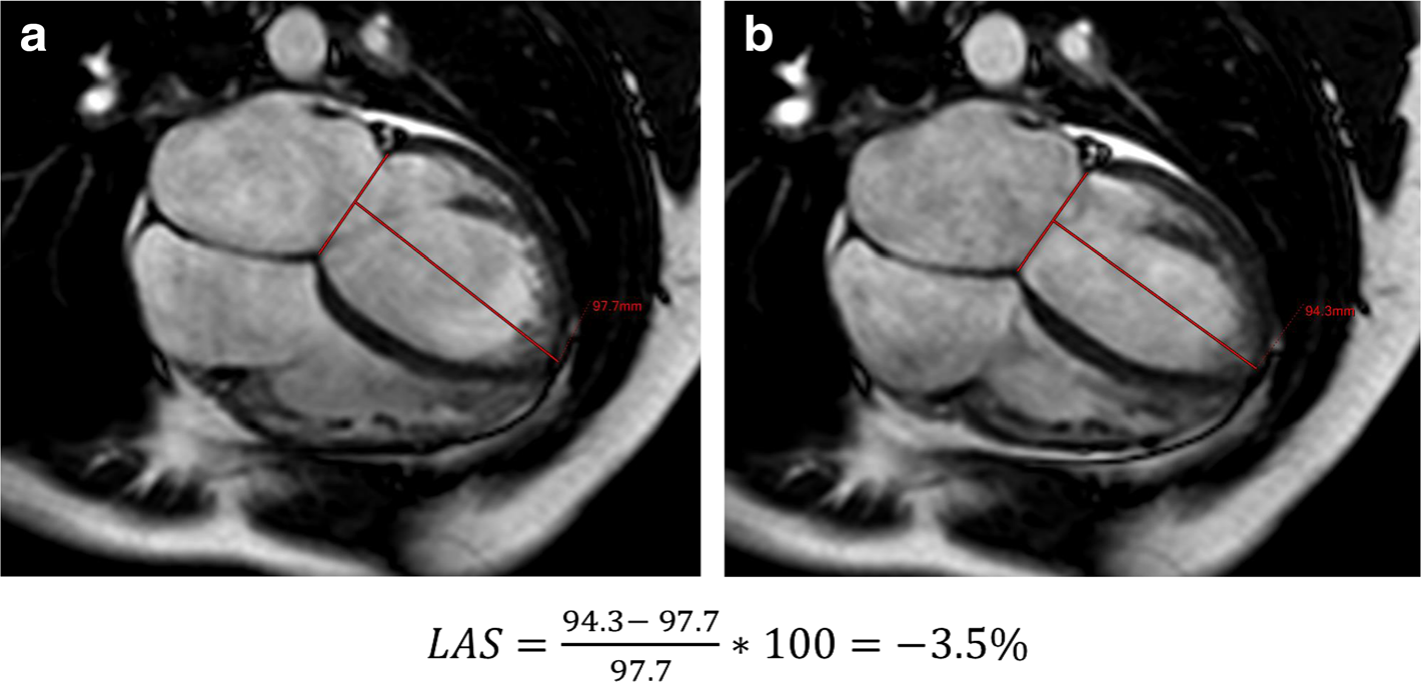
Representative image illustrating the technique for assessment LAS in a patient with severe NIDCM in end-diastole (**a**) and end-systole (**b**)

Mean values in 2- and 4-chamber views were calculated. All values were assessed with the IntelliSpace Portal (ISP) workspace (Version 6, Philips Healthcare, Best, the Netherlands). An example of the technique in a patient with a severe NIDCM is shown in Fig. 1. The technique has been described and validated in detail previously [20].

### Intra- and interobserver variability

For calculation of intra- and interobserver variability 20 single measurements were used. To examine intraobserver variability, a sample of 20 randomly selected CMR scans for the measurement of LAS were randomly selected for masked review by the same investigator. The same studies were analysed by a co-investigator, who was blinded to the clinical information and the results of the first investigation, in order to measure interobserver variability.

### Statistics

Statistical analysis was carried out using the software solution MedCalc (Version 13.1.2.0, MedCalc Software, Ostend, Belgium). Continuous variables are expressed as mean ± standard deviation. For the comparison of means between groups, two-tailed Student’s *t*-test was used. Proportions of categorical were compared using Chi-squared test. Survival curves were plotted using the Kaplan-Meier method and *p*-values were determined by log-rank testing. The calculation of optimal cut-off values was carried out by receiver operating characteristics (ROC) analysis. Cox proportional hazards models were used for uni- and multivariate analysis, which yielded hazard ratios (HR) and the corresponding 95 % confidence intervals (CI). *P*-values <0.05 were regarded as significantly different. After successful testing for normal distribution with Shapiro-Wilk test, correlation coefficients were expressed as Pearson’s r.

## Results

### Clinical characteristics

One-hundred-forty-six patients were included for data collection. During a follow-up period of 4.3 ± 2.0 years, 24 hard cardiac events occurred (cardiac death, heart transplantation or aborted sudden cardiac death due to appropriate ICD shock delivery), declared as primary endpoint. Further 10 patients were admitted to the hospital due to severe heart failure symptoms during the follow-up period (composite secondary endpoint, *n* = 34). The baseline characteristics of the study population are shown in Table 1. The mean values for all CMR parameters in patients with cardiac events including LAS were significantly different from those in patients without cardiac events (Table 2). The mean values for LAS were −5.1 ± 2.6 % in patients with endpoint and −8.5 ± 3.2 % in patients without endpoint (*p* < 0.0001).

**Table 1:** Baseline characteristics of the study population

Parameter	All patients (*n* = 146)	Patients without endpoint (*n* = 112)	Patients with endpoint (*n* = 34)	*P*-value
Clinical data				
Age (years)	53 ± 14	52 ± 14	57 ± 15	NS
Male gender, *n* (%)	116 (80)	90 (80)	26 (76)	NS
Arterial hypertension, *n* (%)	64 (44)	48 (43)	16 (47)	NS
Hyperlipidaemia, *n* (%)	27 (18)	21 (19)	6 (18)	NS
Smoking, *n* (%)	50 (34)	41 (37)	9 (36)	NS
Diabetes mellitus, *n* (%)	20 (14)	16 (14)	4 (12)	NS
Familiar cardiomyopathy, *n* (%)	14 (10)	13 (12)	1 (3)	NS
Body mass index (kg/m^2^)	25.7 ± 3.9	25.7 ± 3.9	25.5 ± 3.7	NS
NYHA Class				
I, *n* (%)	18 (12)	13 (12)	5 (15)	NS
II, *n* (%)	72 (49)	60 (54)	12 (35)	NS
III, *n* (%)	56 (38)	39 (35)	17 (50)	NS
Laboratory data				
Serum creatinine (mg/dL)	1.12 ± 1.00	1.13 ± 1.13	1.07 ± 0.35	NS
Cardiac medications				
β-blockers, *n* (%)	141 (97)	108 (96)	33 (97)	NS
ACE-Inhibitors/AT II blockers, *n* (%)	143 (98)	109 (97)	34 (100)	NS
Spironolactone, *n* (%)	79 (54)	61 (54)	18 (53)	NS
Diuretics, *n* (%)	91 (62)	67 (60)	24 (71)	NS
Digoxin, *n* (%)	31 (21)	18 (16)	13 (38)	<0.05
Coumadine, *n* (%)	61 (42)	44 (39)	17 (50)	NS

**Table 2:** CMR parameter

Parameter	All patients (*n* = 146)	Patients without endpoint (*n* = 112)	Patients with endpoint (*n* = 34)	*P*-value
LVEDV (mL)	291 ± 102	276 ± 88	341 ± 127	0.05
LVEDV/BSA (mL/m^2^)	148 ± 50	140 ± 41	175 ± 66	<0.0001
LVESV (mL)	214 ± 104	195 ± 86	275 ± 132	<0.0001
LVESV/BSA (mL/m^2^)	108 ± 52	98 ± 41	143 ± 69	<0.0001
LVEF (%)	29.3 ± 11.0	31.5 ± 10.1	22.0 ± 10.9	<0.0001
LVGFI (%)	20.3 ± 8.0	22.0 ± 7.5	14.8 ± 7.2	<0.0001
Cardiac output (L/min)	5.5 ± 1.6	5.6 ± 1.6	5.1 ± 1.7	NS
Cardiac output index (L/min*m^2^)	2.8 ± 0.8	2.9 ± 0.7	2.6 ± 0.8	<0.05
LGE present, *n* (%)	64 (44)	44 (39)	20 (59)	<0.05
LAS (%)	−7.7 ± 3.4	−8.5 ± 3.2	−5.1 ± 2.6	<0.0001

### Intra- and interobserver variability

Intra- and interobserver variability for single measurements of LAS were low with 5.1 ± 3.8 % and 5.3 ± 3.9 %, respectively.

### Survival analysis

**Fig. 2:**
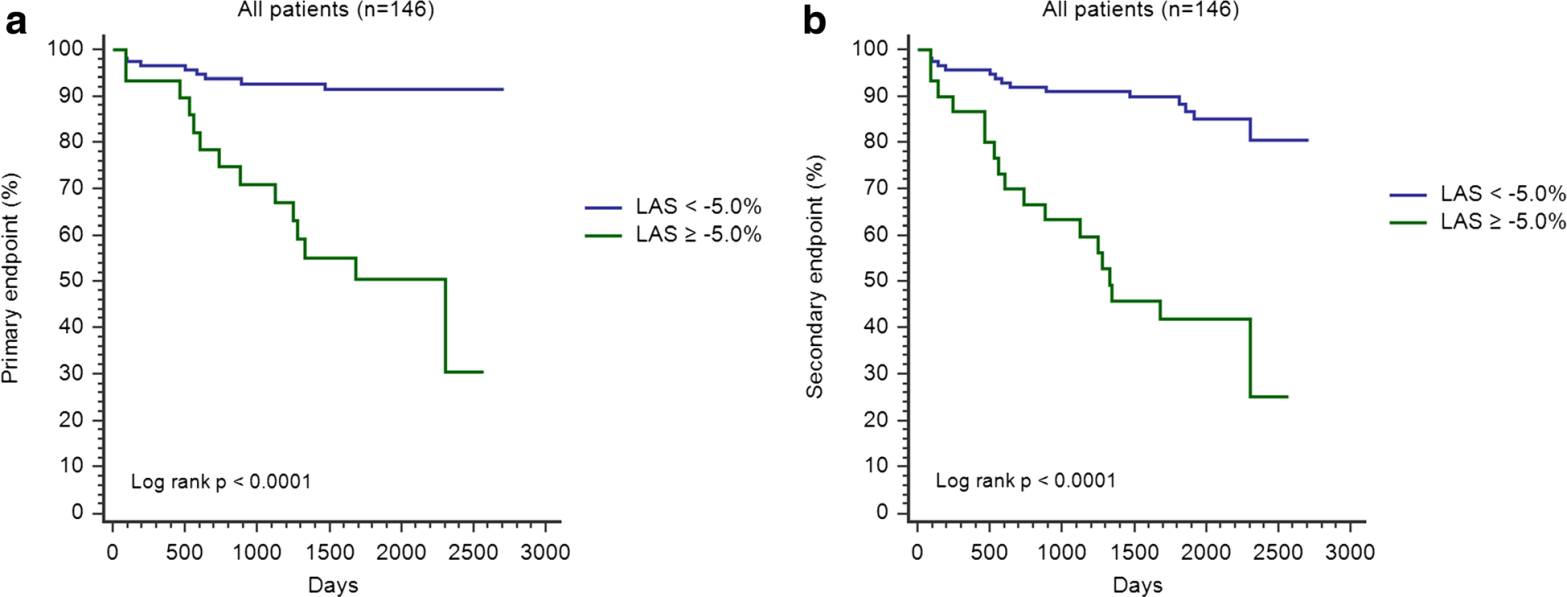
Kaplan-Meier curves including all patients (*n* = 146) for the primary (**a**) and secondary (**b**) endpoint (LAS cut-off value: −5.0 %)

**Fig. 3:**
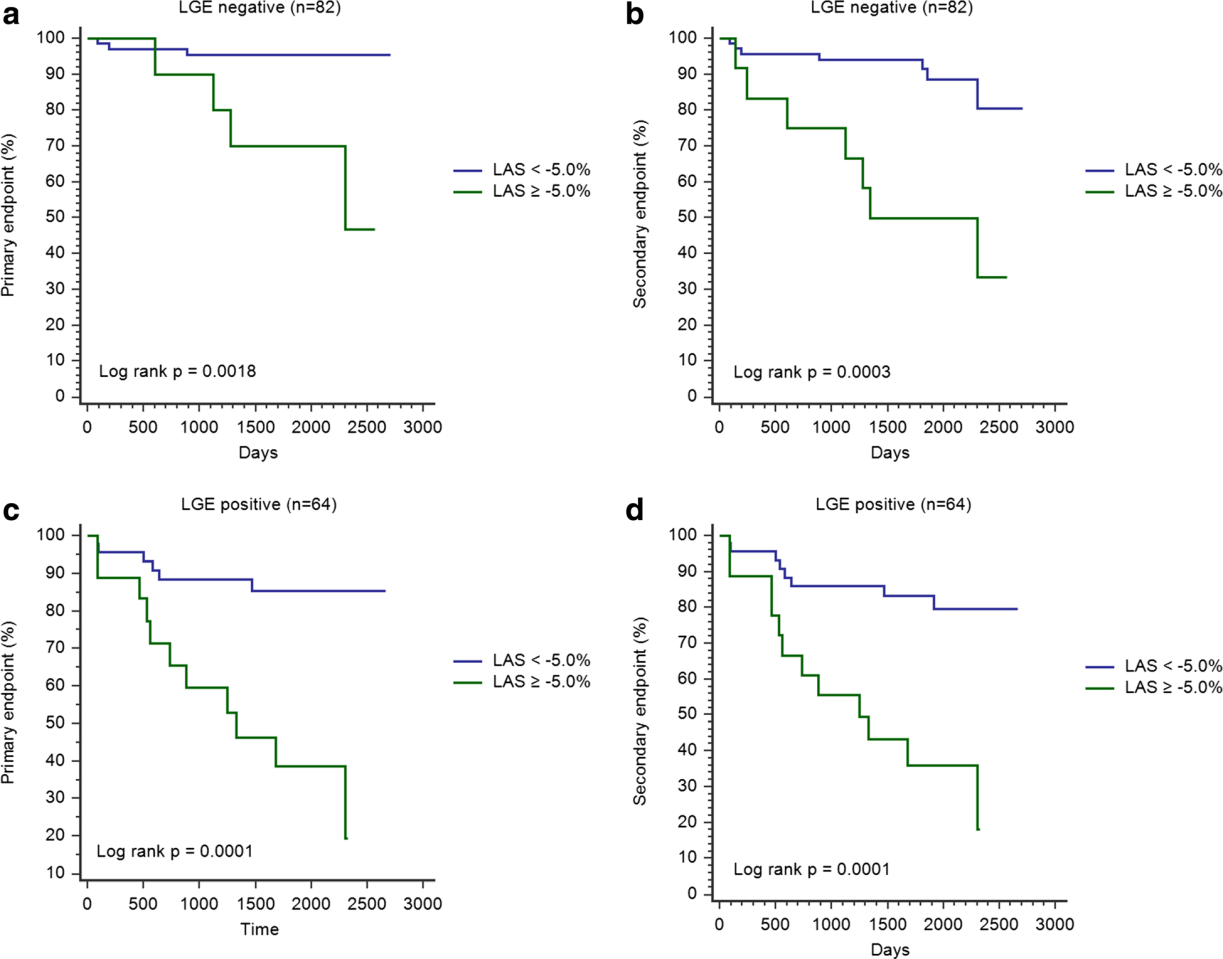
Kaplan-Meier curves for the primary (**a**+**c**) and secondary (**b**+**d**) endpoint including patients with and without LGE (LAS cut-off value: −5.0 %)

ROC analysis yielded a LAS cut-off value of −5.0 % for the primary endpoint (AUC of 0.81 sensitivity of 62.5 %, specificity of 88.5 %). Kaplan-Meier curves for the primary and secondary endpoint concerning the whole study cohort are shown in Fig. 2. Patients with LAS of −5.0 % or worse showed a significantly higher rate of cardiac events. For both, the primary and secondary endpoint, a LAS cut-off value of −5.0 % was highly prognostic for the prediction of cardiac events in patients with or without LGE (Fig. 3).

### Uni- and multivariate analysis and comparison to LVEF and LGE

**Fig. 4:**
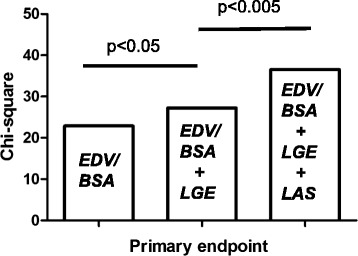
Incremental predictive value of LAS regarding the primary endpoint. In this multivariate model we started with entering the LVEDV/BAS, followed by adding LGE and LAS. LAS offered gradual prognostic information to the model (Table 5)

Table 3 shows the results of univariate analysis regarding clinical and CMR data. According to the analysis, age, LVEF, left ventricular end-diastolic volume (LVEDV)/body surface area (BSA), the presence of LGE, LVGFI and LAS were univariate predictors of cardiac events. In a stepwise multivariate Cox regression analysis including the LVEF, the presence of LGE, LVGFI, LVEDV/BSA and LAS, LAS (HR: 1.28, *p* = 0.006), LVEDV/BSA (HR: 1.01, *p* = 0.044) and the presence of LGE (HR: 2.51, *p* = 0.046) remained the only significant parameters regarding the primary endpoint (Table 4). LVGFI and LVEF were no longer significant variables in this model. For the secondary endpoint, LAS remained the only independent predictor for adverse events (HR: 1.26, *p* = 0.009), surpassing LVEDV/BSA and LGE. To further test the predictive power of LAS we performed a sequential Cox regression analysis regarding the primary endpoint. Hereby, the addition of LAS to a model including LGE and the LVEDV/BSA led to a significant increase in the predictive power of this model (Fig. 4 and Table 5).

**Table 3:** Univariate analysis of all patients (*n* = 146) for primary and secondary endpoint

Variable	Primary endpoint			Secondary endpoint		
	HR	95%CI	*p*-value	HR	95%CI	*p*-value
Gender	0.91	0.34–2.43	0.85	0.77	0.35–1.70	0.52
Age (yrs)	1.02	0.99–1.05	0.15	1.03	1.00–1.05	**0.04**
NYHA class	1.13	0.60–2.11	0.70	1.32	0.78–2.26	0.31
LVEF (%)	0.90	0.86–0.94	**<0.0001**	0.92	0.89–0.95	**<0.0001**
LVEDV/BSA (ml/m^2^)	1.02	1.01–1.02	**<0.0001**	1.01	1.01–1.02	**<0.0001**
LGE present	3.43	1.42–8.27	**0.006**	2.00	1.01–3.95	**0.048**
LVGFI (%)	0.86	0.81–0.91	**<0.0001**	0.89	0.85–0.93	**<0.0001**
LAS (%)	1.46	1.25–1.71	**<0.0001**	1.41	1.23–1.60	**<0.0001**

Univariate analysis revealed that LVEF, LVEDV/BSA, LGE present, LVGFI and LAS were significant paramters regarding the primary endpoint, while age, LVEF, LVEDV/BSA, LGE present, LVGFI and LAS were significantly associated with the secondary endpoint (significant *p*-values in bold letters)

**Table 4:** Multivariate proportional-hazard model including LAS, LVGFI, LVEDV/BSA, LVEF and LGE (all patients) for primary and secondary endpoint

Variable	Primary endpoint			Secondary endpoint		
	HR	95%CI	*p*-value	HR	95%CI	*p*-value
LVEDV/BSA (ml/m^2^)	1.01	1.00–1.02	**0.044**	1.00	0.99–1.01	0.84
LGE present	2.51	1.02–6.19	**0.046**	1.64	0.82–3.29	0.16
LAS (%)	1.28	1.07–1.52	**0.006**	1.26	1.06–1.50	**0.009**

In the multivariate analysis LVEDV/BSA, LGE present and LAS were significantly associated with the primary endpoint, while LAS was the only significant parameter regarding the secondary endpoint (significant *p*-values in bold letters)

**Table 5:** Comparison of multivariate Cox-regression models including LGE, LVEDV/BSA and LAS

Comparison of multivariate Cox-regression models; *n* = 146			
Model 1	Model 2	Chi^2^ difference	*p*
LVEDV/BSA	LVEDV/BSA (ml/m^2^) + LGE present	4.26	**<0.05**
LVEDV/BSA (ml/m^2^) + LGE present	LVEDV/BSA (ml/m^2^) + LGE present + LAS (%)	9.33	**<0.005**

Cox regression analysis revealed a significant increase in the predictive power when adding LAS to a model including LGE and LVEDV/BSA (significant *p*-values in bold letters)

### Dichotomous prognosis scoring system

**Fig. 5:**
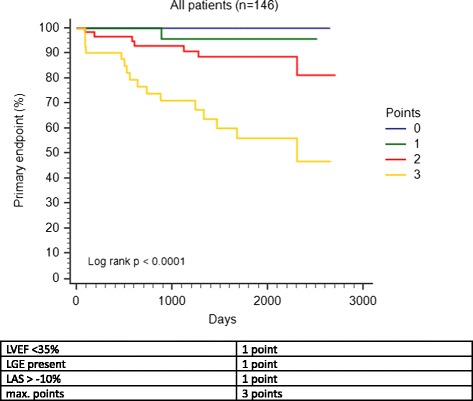
Kaplan-Meier curves based on a dichotomous scoring model. The scoring system ranges from 0 to 3 points: 1 point for LVEF <35 %, 1 point for the presence of LGE and 1 point for LAS > −10 %

For risk stratification in patients with NIDCM exclusively with CMR, we created a three point scoring model including LVEF, the presence of LGE and LAS using the following criteria: LVEF <35 %, LGE present and LAS > −10 %. We used a cut-off value for the LVEF <35 % as this is also the recommended cut-off value for the need of a defibrillator in ischemic cardiomyopathies and for LAS > −10 % as there was no single cardiac event in patients with LAS values better than −10 % in our patient collective. The scoring system and the resulting Kaplan-Meier curves can be found in Fig. 5. We observed that patients with 3 points had significant higher rates of hard cardiac events during the follow-up period than those with 2 or fewer points.

## Discussion

LAS has been shown to be a simple and rapidly assessable parameter representing global LV longitudinal function without necessity of post-processing software tools [20]. In the present study we evaluated for the first time the prognostic value of LAS in patients with NIDCM. As a main finding we observed that LAS was significantly associated with hard cardiac events in NIDCM patients with or without appearance of LGE. More importantly, a dichotomous scoring system including standard CMR parameters and LAS may help to identify high-risk patients with NIDCM. Patients with 3 points (LVEF <35 %, presence of LGE and LAS > −10 %) had a probability for cardiac events over 50 %, while in patients with 0 points no event occurred during the follow-up period.

In patients with suspected heart failure an early diagnosis, a precise risk stratification and the detection of adverse cardiac remodelling is essential for early therapeutic intervention and reduction of mortality [23]. Due to its excellent intrinsic blood-to-tissue contrast and high reproducibility, CMR has emerged as an accurate method for the evaluation of left ventricular function, which can be used for cost-effective and accurate diagnostic classification of patients with heart failure [11].

The complex myocardial deformation behaviour can be characterized by means of longitudinal, circumferential and torsional parameters. The measurement of LVEF alone gives an incomplete representation of the cardiac function [24]. Despite several studies which emphasize the incremental value of strain imaging for the detection of ischemic myocardium and for the risk stratification of patients with ischemic heart disease and cardiomyopathies [16, 17, 19, 25], important clinical decision makings e.g. regarding implantation an ICD, initiate cardiac resynchronization therapy or adjust medical treatment are often based on cardiac function as assessed by the LVEF [26–28].

Echocardiographic studies showed that measurement of LVEF has high observer variability and poor agreement with other methods [29–31]. Particularly quantification of longitudinal strain may therefore be a better measure of contractile cardiac function than calculating the LVEF [32]. Carlsson et al. analyzed longitudinal atrio-ventricular plane displacement with CMR in normal subjects, professional athletes and patients with dilated cardiomyopathy and reduced LVEF (<30 %). They could show that the percentage of the stroke volume generated by longitudinal function was similar in all groups at about 60 % even though stroke volume was higher in athletes and lower in patients with cardiomyopathy [33].

Several studies support our hypothesis that longitudinal function assessment is a better indicator for prognosis in various cardiovascular diseases [34–37]. In a prospective study of Sveälv et al. longitudinal function assessed with echocardiography remained an independent prognostic variable for survival in NIDCM patients, emphasising the importance of the basal segments of the ventricles for ventricular function [38]. Other groups reported similar data on heart failure patients (ischaemic and non-ischaemic cardiomyopathy) conforming that the estimation of longitudinal function by strain imaging is a useful surrogate of all-cause mortality [39, 40]. However, most of these data are based on echocardiographic assessment of cardiac deformation.

In a recently published CMR study Gjesdal et al. analyzed LAS and its association with outcome in the Multi-Ethnic Study of Atherosclerosis (MESA). In this study, LAS was measured in a similar approach as previously described by us and was found to be a significant predictor for cardiac events in a multi-ethnic population [41]. Lately, we could show that FTI derived longitudinal strain is an independent predictor of survival in patients with NIDCM and offers incremental information for risk stratification beyond clinical parameters, biomarker, and standard CMR [19]. Motoki et al. observed in patients with systolic heart failure that global longitudinal strain assessed with echocardiography was the most robust strain parameter for risk stratification of patients with non-ischemic heart failure and provides incremental prognostic value to the LVEF [16]. A meta-analysis of Kalam et al. confirmed the strong prognostic value of global longitudinal strain (GLS) determined by echocardiography in different cardiovascular diseases and concluded that GLS may even be superior in predicting major cardiovascular events than the LVEF [42]. This is in line with our findings as CMR-derived LAS is significantly associated with outcome in NIDCM patients.

Compared to the studies mentioned above we evaluated a parameter which has several advantages particularly for usage in clinical routine. Major drawbacks of FTI strain analysis are the need of a specific post-processing software tool and an elaborate manual marking of the myocardial borders. By contrast, even in clinical routine LAS can be measured rapidly based on standard SSFP sequences. Not only being a readily available parameter, our study could show that LAS has significant prognostic value in patients with NIDCM. LAS values > −5.0 % were significantly associated with a higher rate of cardiac events irrespectively of the presence of LGE. This is in concordance with our previous study on FTI where we could show that longitudinal strain surpasses LGE in predicting outcome [19]. LAS may therefore be a safe and cheap alternative to LGE imaging for risk stratification. Interestingly, in our multivariate analysis LVEDV/BSA, LGE and LAS were significantly associated with outcome, while LVEF and LVGFI were no longer significant variables. On the one hand this may be due to the fact that LVEF and LVGFI have a high correlation (*r* = 0.97), on the other hand the patients in our collective have low LVEF values (29.3 ± 11.0 %), which may also explain why LVEF is not as strong as in other studies.

Regarding LAS, we observed no single cardiac event in patients with LAS values better than −10 %. Based on this information we created a scoring system for risk stratification based on LAS and well-established, standard CMR parameters. We decided to use LVEF and not LVEDV/BSA for our risk stratification model because LVEF is still the most established parameter for risk stratification in patients with NIDCM and in a multivariate model including LVEF, LAS and LGE significantly associated with the endpoint (data not shown). We believe that for clinical routine LVEF may be better applicable than LVEDV/BSA.

Hereby, we observed that detection of any LGE, LVEF below 35 % and LAS > −10 %, while each feature counted as one point in our scoring system, is highly associated with cardiac events. In patients without these risk features (score = 0) no events were observed during the follow-up period. Although, several studies suggest that longitudinal function outperforms the LVEF in predicting survival in cardiomyopathies [42], we here propose a combined risk index for improved risk stratification consisting of LVEF, LGE and LAS.

### Limitations

LAS was analyzed retrospectively. LAS does exclusively represent LV longitudinal strain and does not provide information about strain rate, intra-myocardial strain or circumferential and radial strain, which may better reflect cardiac function in certain circumstances. Apart from LAS, no further strain measurements were performed. Extracellular volume could not be analyzed, as T1 mapping was not available in this study.

Moreover, LAS was exclusively evaluated in NIDCM patients with EF below 45 %. The applicability of LAS on patients with mild form of NIDCM has not been evaluated and has to be tested in further studies.

## Conclusion

LAS is a rapidly determinable parameter which can be assessed easily from routinely acquired, contrast-agent free cine SSFP sequences. A reduced LAS is significantly associated with the occurrence of cardiac events. Accordingly, LAS is of high prognostic value in patients with NIDCM besides LGE and LVEDV/BSA. The scoring model presented in this work, which combines LVEF and LGE - the most established CMR parameters- and LAS, may help to improve risk stratification in NIDCM.

## Abbreviations

NIDCM, non-ischemic dilated cardiomyopathy; LVEF, left ventricular ejection fraction; CMR, cardiovascular magnetic resonance; LGE, late gadolinium enhancement; FTI, feature tracking imaging; LAS, long axis strain; ICD, implantable cardioverter defibrillator; SCD, sudden cardiac death; SSFP, steady state free precession; LVGFI, left ventricular global function index; ISP, IntelliSpace Portal; ROC, receiver operating characteristics; HR, hazard ratio; CI, confidence interval; AUC, area under the curve; LVEDV, left ventricular enddiastolic volume; BSA, body surface area; GLS, global longitudinal strain
